# An Optical Model for Translucent Volume Rendering and Its Implementation Using the Preintegrated Shear-Warp Algorithm

**DOI:** 10.1155/2010/429051

**Published:** 2010-05-31

**Authors:** Bin Li, Lianfang Tian, Shanxing Ou

**Affiliations:** ^1^School of Automation Science and Engineering, South China University of Technology, Guangzhou, Guangdong 510640, China; ^2^Department of Radiology, Guangzhou General Hospital of Guangzhou Command, Guangzhou, Guangdong 510010, China

## Abstract

In order to efficiently and effectively reconstruct 3D medical images and clearly display the detailed information of inner structures and the inner hidden interfaces between different media, an Improved Volume Rendering Optical Model (IVROM) for medical translucent volume rendering and its implementation using the preintegrated Shear-Warp Volume Rendering algorithm are proposed in this paper, which can be readily applied on a commodity PC. Based on the classical absorption and emission model, effects of volumetric shadows and direct and indirect scattering are also considered in the proposed model IVROM. Moreover, the implementation of the Improved Translucent Volume Rendering Method (ITVRM) integrating the IVROM model, Shear-Warp and preintegrated volume rendering algorithm is described, in which the aliasing and staircase effects resulting from under-sampling in Shear-Warp, are avoided by the preintegrated volume rendering technique. This study demonstrates the superiority of the proposed method.

## 1. Introduction

In medical visualization applications, such as computer aided diagnosis and therapy, the relative position between the tumor and its adjacent tissues is often determined by physicians through observing the inner pathologic changes of tissue structures and the detailed information of the tissue surface, before making a reasonable therapy planning. The detailed information of inner structures and inner hidden interfaces between different layers can be clearly displayed by using the translucent volume rendering technique, which has been widely applied in medical visualization.

Lighting Absorption and Emission Model combined with Blinn-Phong surface shading model (LAEM-BP) is one of the most widely used lighting models in direct volume rendering in medical visualization [1, 2]. The optical model was described in papers by Sabella [3], Drebin et al. [4], and Levoy [5], in which the volume shading incorporating diffuse and specular shading by approximating the surface normal with the gradient of the 3D field is described, but scattering is ignored in order to achieve fast approximation used by direct lighting. Moreover, in order to realize interactive volume rendering of 3D scalar fields of medical data, the optical model as well as its volume rendering method is implemented in hardware [6, 7]. The model can approximately render obvious translucent tissue surface where the normal is well defined for regions in the volume that have high gradient magnitudes. However, this method cannot clearly display the detailed information of inner structures and the inner hidden interfaces between different media, especially when the scattering becomes the main factor that affects the rending effects and dominates the visual appearance [2]. 

The effect of multiple scattering and indirect illumination is important for volume rendering applications [1, 2, 8, 9]. Blinn [10] presents a model for the reflection and transmission of light through thin clouds of particles based on probabilistic arguments and single scattering approximation. Kajiya and Von Herzen describe a model for rendering arbitrary volume densities that includes expensive multiple scattering computation [11]. In their methods, the radiative transport equation [12] is employed, and expensive and sophisticated numerical methods must also be employed to compute the radiance distribution to a desired accuracy.

Furthermore, Max et al. [13] use a one-dimensional scattering equation to calculate the light transport, where forward peaked phase function as the hemisphere is needed to be discretized finely. Spherical harmonics are also used by Kajiya and Von Herzen [11] to calculate anisotropic scattering. Monte Carlo methods are robust and simple techniques for solving light transport equation. Hanrahan and Krueger model scattering in layered surfaces with linear transport theory and derive explicit formulas for backscattering and transmission [14]. Pharr and Hanrahan describe a mathematical framework [15] to solve the scattering equation in context of a variety of rendering problems and also give a numerical Monte Carlo sampling method. The abovementioned models are both powerful and robust but will suffer from problems coming from standard Monte Carlo method such as slow convergence and noise. To solve such problems, Stam and Fiume show that the widely used diffusion approximation can produce good results for scattering in dense media [16]. Jensen et al. introduce a computationally efficient and analytical diffusion approximation method for multiple scattering [9], which is especially preferable for homogeneous materials that exhibit considerable subsurface light transport ability; however, the model does not appear to be easily extended to volumes with arbitrary optical properties [2].

As a matter of fact, simulations about full light transport are computationally expensive and interactive operation is limited; for example, the change of the illumination or transfer function cannot executed on-line. On the other hand, although there exist analytical approximation methods, most of them are derived based on some assumptions, such as homogeneous optical properties, density, simple lighting, and unrealistic boundary conditions [2]. So such analytical approximation models cannot be used for arbitrary volume rendering or scanned data where optical properties such as absorption and scattering coefficients are hard to be obtained. The disadvantages of huge computation cost and large memory requirement hamper its application in practice. Therefore, some optical empirical models that are relatively easy to implement in medical volume rendering are widely used in medical applications. Those optical empirical models can be realized by graphics hardware-based or CPU-based volume rendering methods. For example, based on the method in document [1], graphics hardware-based optical empirical models for direct volume rendering are developed in documents [2, 17–20]. As to CPU-based method, it is well known that the Shear-Warp method [21] is currently the fastest CPU-based volume rendering method [22, 23], of which the Shear-Warp shell rendering method [24] is proven to be extremely fast. Besides that, some other empirical optimal models are also in common use; for example, the basic lighting absorption and emission model is modified by adding a direct scattering factor to enhance the display effect of inner hidden interfaces.

Shear-warp is considered to be the fastest software algorithm, but it achieves this rendering speed only by sacrificing interpolation between the slices of the volume data. The resample method in traditional Shear-Warp algorithm leads to aliasing and staircase effects, which is in general insufficient for medical purposes. Preintegrated volume rendering [9] provides an efficient way to interpolate in-between slices of the volume data with some loss in rendering performance [23]. Document [23] integrates preintegrated volume rendering into the Shear-Warp algorithm to overcome its drawback. But how to integrate the lighting model into preintegrated volume rendering has not been considered elaborately in document [23].

In this study, an improved volume rendering optical model (IVROM) for medical translucent volume rendering and its implementation using the Shear-Warp and preintegrated Volume Rendering algorithm are proposed in this paper, which can be readily applied on a commodity PC. In the proposed model, the lighting absorption and emission model are employed; moreover, other factors are also considered such as the volumetric shadowing, direct, and indirect emission. Furthermore, the display effect of inner hidden interfaces is enhanced by employing rendering technique of feeling of unreality. Finally, the realization of Improved Translucent Volume Rendering Method (ITVRM) combining the IVROM model, Shear-Warp, and preintegrated volume rendering algorithm is described in detail, in which the aliasing and staircase effects resulting from under-sampling in traditional Shear-Warp algorithm are avoided by the preintegrated volume rendering. By using the proposed method, the 3D medical image can be reconstructed efficiently, and the detailed information of inner structures, as well as the inner hidden interfaces between different layers, can be clearly displayed.

## 2. The Description of LAEM-BP

Important terms used in the paper are found in Table 1.

Classical lighting absorption and emission model is described as follows:

(1) $$I\left(x_{1},\vec{\omega }\right)=T\left(0,l\right)I\left(x_{0},\vec{\omega }\right)+\int_{0}^{l}T\left(s,l\right)*R\left(x\left(s\right)\right)ds,$$

where *x*(*s*) is the 3D coordinate at position *s* along the view direction. *T*(*s*, *l*) is the attenuation from *x*(*s*) to *x*(*l*) along the view direction, *T*(*s*, *l*) = exp (−∫_*s*_^*l*^ *τ*(*t*)*d* *t*).

Usually, the scattering lighting intensity at position x along $\vec{\omega }$ direction is

(2) $$S\left(x,\vec{\omega }\right)=r\left(x,\vec{\omega },\omega^{\prime }\right)I\left(x,\omega^{\prime }\right),$$

where *I*(*x*, *ω*′) is the lighting intensity at position x along the ray direction *ω*′. $r\left(x,\vec{\omega },\omega^{\prime }\right)$ is a bidirectional reflection distribution function (BRDF).

In order to realize surface shading, Blinn-Phong model is used, which depicts the part of the direct scattering of $r(x,\vec{\omega },\omega^{\prime })$. If there is no obstacle along the ray direction *ω*′, *I*(*x*, *ω*′) is equal to the intensity of light source *L*_*g*_. Therefore, combining the Blinn-Phong surface shading model with (1), the LAEM-BP can be written as

(3) $$\begin{array}{cc} I\left(x_{1},\vec{\omega }\right) & =T\left(0,l\right)I\left(x_{0},\vec{\omega }\right)\\ & \;+\int_{0}^{l}T\left(s,l\right)*R\left(\text{x}\left(s\right)\right)*B_{s}\left(x\left(s\right)\right)*L_{g}ds, \end{array}$$

where *B*_*s*_(*x*(*s*)) is the value of Blinn-Phong surface shading model which is approximated by the normalized gradient at position *x*(*s*); *s* denotes the distance from the view point to some point along the view direction; *l* is the length from the view point to some point along the view direction; *L*_*g*_ denotes light source intensity; subscript *g* denotes ray direction along *ω*′.

## 3. The IVROM Optical Model

The LAEM-BP is more suitable for obvious translucent tissue surface; however, it cannot clearly display the detailed information of the inner structures and the inner hidden interfaces between different mediums, especially when scattering is the dominant factor in the model. Also, it cannot clearly display the region with low gradient yet belong to different tissues. Because a ray has different attenuation values for different tissues, the intershading can be used to compensate the shortcoming existent in LAEM-BP.

In reality, there exists attenuation phenomenon when a ray goes through the tissue. Suppose that the lighting intensity of the indirect ray along *ω*′ direction whose source intensity is *L*_*g*_ at position *x* is *I* ^*i*^(*x*, *ω*′). Then

(4) $$\begin{array}{c} I^{i}\left(x,\omega^{\prime }\right)=L_{g}T_{g}\left(s,t^{\prime }\right),\\ T_{g}\left(s,t^{\prime }\right)=\exp\;\;\left(-\int_{0}^{t\prime }\tau \left(x\left(s\right)+{\vec{\omega }}_{l}t\right)dt\right). \end{array}$$

So, (3) can be rewritten as

(5) $$\begin{array}{cc} I\left(x_{1},\vec{\omega }\right) & =T\left(0,l\right)I\left(x_{0},\vec{\omega }\right)\\ & \;+\int_{0}^{l}T\left(s,l\right)*R\left(x\left(s\right)\right)*B_{s}\left(x\left(s\right)\right)*L_{g}*T_{g}\left(s,l_{g}\right)ds, \end{array}$$

where *l*_*g*_ denotes the distance along ray direction, and subscript *g* denotes ray direction along *ω*′. In a randomly distributed media space *V*, when a ray goes through the space *V*, there exists extinction phenomenon of absorption and scattering along the transmission direction due to the interaction between the light and medium particles. Therefore, $I(x,\vec{\omega })$ at position *x* should be considered to include the collimation radiation intensity and scattering radiation intensity. Furthermore, the latter part can be divided into two parts: the direct scattering and indirect scattering. Assume that $r(x,\vec{\omega },\omega^{\prime })$ is a bidirectional reflection distribution function (BRDF); BRDF of a particle at position *x* is

(6) $$r\left(x,\vec{\omega },\omega^{\prime }\right)=a\left(x\right)\tau \left(x\right)p\left(\vec{\omega },\omega^{\prime }\right),$$

where *a*(*x*) is the reflectance ratio of a particle; $p^{d}(\vec{\omega },\omega^{\prime })$ is phase function, which denotes its direction of scattering.

In fact, in (3), the effect of direct scattering has to be considered. Let $p^{d}(\vec{\omega },\omega^{\prime })$ denote the phase function of direct scattering; then

(7) $$p^{d}\left(\vec{\omega },\omega^{\prime }\right)=\left|\vec{N}\cdot \omega^{\prime }\right|+{\left(\vec{N}\cdot \frac{\vec{\omega }+\omega^{\prime }}{\left|\vec{\omega }+\omega^{\prime }\right|}\right)}^{n},$$

where $\vec{N}$ is the gradient at position *x*; in other word, it is the normal vectors of the inner hidden interfaces between different mediums. Considering positive and reverse direction of hidden interfaces, the absolute value of $\left|\vec{N}\cdot \omega^{\prime }\right|$ is given.

If the gradient value in a region is high, the Blinn-Phong surface shading model is used to calculate shading effect; reversely, if the gradient value of the region is low, the above model is used to realize shading computation. Therefore, (5) can be rewritten as

(8) $$\begin{array}{cc} I\left(x_{1},\vec{\omega }\right) & =T\left(0,l\right)I\left(x_{0},\vec{\omega }\right)\\ & \;+\int_{0}^{l}T\left(s,l\right)*S\left(s\right)*L_{g}*T_{g}\left(s,l_{g}\right)ds, \end{array}$$

(9) $$\begin{array}{cc} S\left(s\right) & =R\left(s\right)\left(\left(1-w\left(s\right)\right)+w\left(s\right)p^{d}\left(\vec{\omega },\omega^{\prime }\right)\right), \end{array}$$

where non-photorealistic rendering is used to enhance the display effect of inner hidden interfaces. For example, a weight can be given for the direct and indirect scattering. Suppose direct scattering *S* ^*d*^(*s*) = *w* ^*d*^ *S*(*s*), where *w* ^*d*^ is the given weight. Obviously, the further the distance to inner hidden interfaces is, the bigger the surface scattering intensity is, and vice versa.

As a fact, the weight function is essentially a boundary detection function; then let weight value *w* ^*d*^ be proportional to the gradient modulus value. Equation (8) can be rewritten as

(10) $$\begin{array}{cc} I\left(x_{1},\vec{\omega }\right) & =T\left(0,l\right)I\left(x_{0},\vec{\omega }\right)\\ & \;+\int_{0}^{l}T\left(s,l\right)*S^{d}\left(s\right)*L_{g}*\exp\;\;\left(-\int_{s}^{l_{g}}\tau \left(t\right)dt\right)ds. \end{array}$$

Next, the effect of indirect scattering is considered. Let indirect scattering be written as

(11) $$S^{i}\left(s\right)=w^{i}R\left(s\right)p^{i}\left(\vec{\omega },\omega^{\prime }\right),$$

where *w* ^*i*^ is the weight, and $p^{i}(\vec{\omega },\omega^{\prime })$ is [25] as follows:

(12) $$p^{i}\left(\vec{\omega },\omega^{\prime }\right)\approx \frac{1}{\sigma \left(x\right)}\sum_{p=1}^{n}B\left(x,{\vec{\omega }}_{p}^{\prime },\vec{\omega }\right)\frac{\Delta \Phi_{p,i}\left(x,{\vec{\omega }}_{p}^{\prime }\right)}{\left(4/3\right)\pi r^{3}},$$

where ΔΦ_*p*,*i*_ is the flux the total lighting intensity carried by some photons that correspond to the indirect illumination. (4/3)*π* *r* ^3^ is the volume of the sphere containing these photons.

Equation (10) is

(13) $$\begin{array}{cc} & I\left(x_{1},\vec{\omega }\right)\\ & \;=T\left(0,l\right)I\left(x_{0},\vec{\omega }\right)\\ & \;\;+\int_{0}^{l}T\left(s,l\right)*(S^{d}\left(s\right)*L_{g}*\exp\;\left(-\int_{s}^{l_{g}}\tau \left(t\right)dt\right)\\ & \;\;\;\;\;\;\;\;\;+S^{i}\left(s\right)*L_{g}*T_{i}\left(s,l_{g}\right))ds, \end{array}$$

where *τ*_*i*_ is the indirect lighting attenuation coefficient, which is dependent on propagation medium. *T*_*i*_(*s*, *l*) denotes the indirect attenuation along a ray from position *x*(*s*) to *x*(*l*), *T*_*i*_(*s*, *l*) = exp (−∫_*s*_^*l*^ *τ*_*i*_(*t*)*d* *t*).

## 4. ITVRM Integrating the IVROM Model, Shear-Warp, and Preintegrated Volume Rendering

### 4.1. Drawback of Shear-Warp

**Figure 1:**
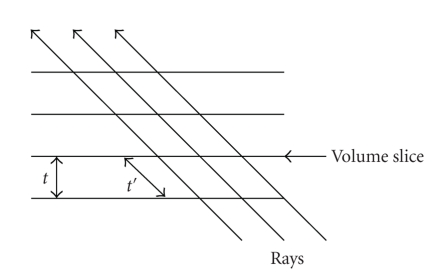
Illustration of resampling in Shear-Warp algorithm.

Due to the in-slice sampling of Shear-Warp, the interslice sampling rate varies depending on the viewing angle [26]. Consider Figure 1 where the 2D case is shown. At 45° the distance *t*′ between adjacent sampling points is $\sqrt{2}t$; on views down a major diagonal it is $\sqrt{3}t$. Basic rules of sampling theory tell that it needs at least a distance of *t* to combat aliasing artifacts and to remain within the Nyquist limit of the sampled signal.

### 4.2. Avoiding Drawback of Shear-Warp by Preintegrated Volume Rendering

Preintegrated volume rendering overcomes the necessity for extremely high sampling rates by splitting the numerical evaluation of the volume rendering integral into two integrations: one for the continuous scalar field and one for transfer functions; thus, the problematic product of Nyquist frequencies as said in Section 4.1 is avoided [23].

Specify colors and extinction coefficients for each scalar value *v* of the volume data by transfer functions *c*(*v*), *T*(*v*), and *T*_*i*_(*v*) [19, 23]. Equation (13) can be then rewritten as

(14) $$\begin{array}{cc} & I\left(v\left(x_{1},\vec{\omega }\right)\right)\\ & \;=T\left(v\left(0,l\right)\right)I\left(v\left(x_{0},\vec{\omega }\right)\right)\\ & \;\;+\int_{0}^{l}T\left(v\left(s,l\right)\right)*(S^{d}\left(s\right)*L_{g}*T\left(v\left(s,l_{g}\right)\right)\\ & \;\;\;\;\;\;\;\;\;\;+S^{i}\left(s\right)*L_{g}*T_{i}\left(v\left(s,l_{g}\right)\right))ds. \end{array}$$

Let *T*(*v*(*s*, *l*)) denote the attenuation along the ray from position *x*(*s*) to *x*(*l*). Let *α* be the opacity of the distance, thus *α* = 1 − *T*(*v*(*s*, *l*)). Similarly, let *α*_*i*_ be the indirect opacity of the distance, thus *α*_*i*_ = 1 − *T*_*i*_(*s*, *l*).

The volume rendering integral (13) can be approximated by a Riemann sum of *n* equal ray segments of length *t* : = *D*/*n*. Thus, the direct opacity *α*_*k*_ of the *k*th segment along the ray is approximated by

(15) $$\alpha_{k}\approx 1-\exp\;\;\left(-\int_{0}^{1}\tau \left(\left(1-\omega \right)v_{f}+\omega v_{b}\right)\right)t\text{d}\omega ,$$

where *v*_*f*_, *v*_*b*_ are the scalar value at the start and the end of the segment *t* along the ray, respectively. Thus, *α*_*k*_ is a function of *v*_*f*_, *v*_*b*_, and *t*, if the latter is not constant. Let *α*_*k*_ = *α*(*v*_*f*_, *v*_*b*_, *t*);

(16) $$\alpha \left(v_{f},v_{b},t\right)\approx 1-\exp\;\;\left(-\frac{t}{v_{b}-v_{f}}\left(T\left(v_{b}\right)-T\left(v_{f}\right)\right)\right),$$

where

(17) $$T\left(v\right)=\int_{0}^{v}\tau \left(v\right)dv.$$

In the same way, let *α*_*ik*_ = *α*_*i*_(*v*_*f*_, *v*_*b*_, *t*); the indirect opacity *α*_*ik*_ of the *k*th segment along the ray is approximated by

(18) $$\alpha_{i}\left(v_{f},v_{b},t\right)\approx 1-\exp\;\left(-\frac{t}{v_{b}-v_{f}}\left(T_{i}\left(v_{b}\right)-T_{i}\left(v_{f}\right)\right)\right),$$

where

(19) $$T_{i}\left(v\right)=\int_{0}^{v}\tau_{i}\left(v\right)dv.$$

The intensity at position x along the view direction $\vec{\omega }$ consists of the colors value of direct lighting and that of indirect lighting. The colors value *C*_*k*_ of direct lighting of the *k*th segment along the ray are approximated correspondingly:

(20) $$\begin{array}{cc} C_{k} & \approx \int_{0}^{1}\tau \left(\left(1-\omega \right)v_{f}+\omega v_{b}\right)c\left(\left(1-\omega \right)v_{f}+\omega v_{b}\right)\\ & \;*S^{d}\left(\left(1-\omega \right)v_{f}+\omega v_{b}\right)\\ & \;*\exp\;\;\left(-\int_{0}^{\omega }\tau \left(\left(1-\omega^{\prime }\right)v_{f}+\omega^{\prime }v_{b}\right)\right)t\text{d}\omega . \end{array}$$

Analogously to *α*_*k*_, *α*_*ik*_, *C*_*k*_ is also a function of *v*_*f*_, *v*_*b*_, and *t*. Let $C_{k}=\tilde{C}(v_{f},v_{b},t)$,

(21) $$\tilde{C}\left(v_{f},v_{b},t\right)\approx \frac{t}{v_{b}-v_{f}}\left(K\left(v_{b}\right)-K\left(v_{f}\right)\right),$$

where

(22) $$K\left(v\right)=\int_{0}^{v}\tau \left(v\right)c\left(v\right)S^{d}dv.$$

### 4.3. Implementation of the ITVRM Algorithm

The proposed ITVRM for medical translucent volume rendering can be easily realized on a commodity PC. The implementation of the CPU-based ITVRM algorithm is described as follows.

#### 4.3.1. Setting of the Transfer Function and the Corresponding Look-Up Table

In order to speed up the medical translucent volume rendering algorithm, the look-up table of the indirect opacity *α*_*i*_ and direct opacity *α* is preset when *t* = 1. Similarly, the primary color *c*_0_ can also be preset when *t* = 1. In the proposed method, the term primary color is borrowed form OpenGL terminology in order to denote the color before shading, which is similar to document [19].

#### 4.3.2. Presetting of the Weight of Direct Scattering

As a matter of fact, the nearer the distance from inner hidden interfaces to somewhere is, the larger its surface scattering intensity is, and vice verse. So the weight function is essentially a boundary detection function; hence the weight of direct scattering *w* ^*d*^ is proportional to the gradient modulus value. Furthermore, if the effect of (11) is considered, then the weight of indirect scattering *w* ^*i*^ should be proportional to the voxel scalar value.

#### 4.3.3. Computing Look-Up Table of Preintegration

The preintegration table is computed. That is, *T*(*v*) and *T*_*i*_(*v*) are preset according to (17) and (19), respectively. *K* ^0^(*v*) is preset according to (23):

(23) $$K^{0}\left(v\right)=\int_{0}^{v}\tau \left(v\right)c_{0}\left(v\right)dv.$$

#### 4.3.4. Composition of the Intermediate Image

**Figure 2:**
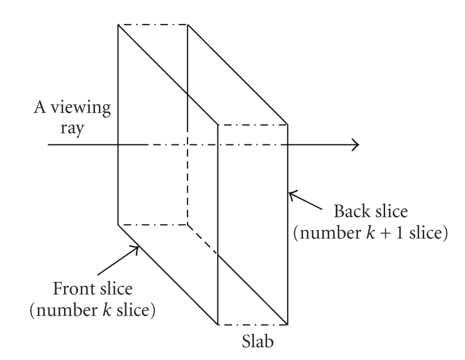
Volume rendering using a slab between two slices.

The proposed ITVRM integrates the IVROM model, Shear-Warp, and preintegrated volume rendering algorithm; so it is similar to the Shear-Warp volume rendering. The implementation of the Shear-Warp volume rendering algorithm is usually divided into two steps: shear transformation of 3D data set and warp of 2D image. The proposed volume rendering method ITVRM renders slabs between adjacent slices instead of individual slices, which is different from the traditional Shear-Warp algorithm, shown in Figure 2. The proposed IVROM will be applied in the composition of the intermediate image in the step of shear transformation of 3D data set. The view direction of 3D discrete data set is usually set arbitrarily by users; so the transformation from object space to image space is also arbitrary. The main idea of Shear-Warp algorithm is that the 3D discrete data set is first transformed into an intermediate coordinate system; then in intermediate coordinate system, the view direction is selected to be parallel to an axis of the coordinate system. Since the direction of light source is arbitrary, without loss of generality, for the convenience of description, the light source is assumed to be in the same side with the view point, which can be judged by whether the included angle is bigger than 90° or not. If the direction of light source is in the other side, then the light composition sequence should be reversed.

The presented ITVRM in the paper applies the IVROM optical model combining Shear-Warp and preintegrated volume rendering algorithm. Similar to the Shear-Warp algorithm, each slab between image slices is processed and composed into intermediate image sequencely from forward to back. For example, when the *k*th slab between the *k*th slice and *k* + 1th slice is processed, the steps are as follows.

**Step 1**

Calculate the direct scattering term; it is given as

(24) $$C_{\text{post}}^{d}=C_{\text{pre}}^{d}+\alpha_{\text{now}}^{d}*C_{\text{now}}^{d}*\left(1-O_{\text{pre}}^{d}\right)*I_{\text{pre}}^{d},$$

where *C*_pre_^*d*^ denotes the current red color component of the RGB value of the pixel at a position of the intermediate image without consideration of direct scattering, and *C*_post_^*d*^ denotes that with direct scattering; *O*_pre_^*d*^ denotes the current opacity value at a position of the intermediate image without consideration of direct scattering; *I*_pre_^*d*^ denotes the accumulative lighting intensity at a position of the intermediate image without consideration of direct scattering; *α*_now_^*d*^ denotes resampling opacity of current processed voxel in the current slab. *C*_now_^*d*^ denotes the R component in current resampling voxel in the current slab. The superscript *d* is for direct scattering. The R component before shading *C*_0 now_^*d*^ in current resampling voxel in the current slab is

(25) $$C_{0\;\text{now}}^{d}=\frac{t}{s_{b}-s_{f}}\left(K_{0}\left(s_{b}\right)-K_{0}\left(s_{f}\right)\right).$$

Then *C*_now_^*d*^ is computed by (22) and (25). So,

(26) $$O_{\text{post}}^{d}=O_{\text{pre}}^{d}+\alpha_{\text{now}}^{d}*\left(1-O_{\text{pre}}^{d}\right),$$

where *O*_post_^*d*^ denotes the opacity value at the current position *p* *o* *s* of the intermediate image after computing direct scattering term.

**Figure 3:**
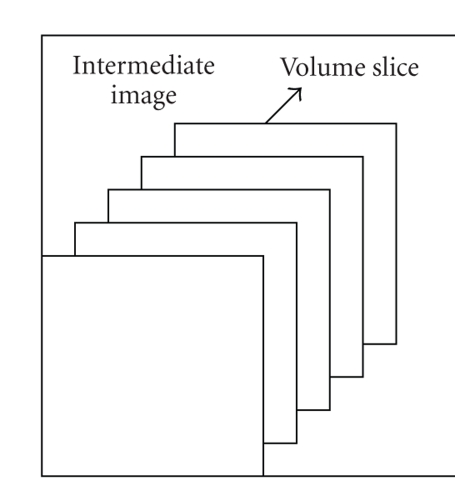
Intermediate image-sized buffer slices.

When the opacity and colors of the current slab are rasampled and composited, the data of back slice data are used as the front slice of the next slab procession which are stored using the intermediate image-sized buffer plane shown as Figure 3.

Since the composition sequence of the light is from back to forward, so

(27) $$I_{\text{post}}^{d}=\left(1-\alpha_{\text{now}}^{d}\right)*I_{\text{pre}}^{d},$$

where *I*_post_^*d*^ denotes the accumulative intensity of direct lighting at current position of the intermediate image with the consideration of direct scattering.

Similarly, the direct scattering value for green and blue color can be calculated in the same way.

**Step 2**

Calculate the accumulative lighting intensity.

Since the composition sequence of the light is from back to forward, so

(28) $$I_{\text{post}}^{i}=I_{\text{pre}}^{i}*\left(1-\alpha_{\text{now}}^{i}\right),$$

where *I*_pre_^*i*^ denotes the current accumulative intensity of indirect lighting at a position of the intermediate image without consideration of indirect scattering. *I*_post_^*i*^ denotes that with indirect scattering. *α*_now_^*i*^ denotes resampling indirect opacity of current voxel. The superscript *i* is for indirect scattering.

**Step 3**

Calculate the indirect scattering term:

(29) $$C_{\text{post}}^{i}=C_{\text{pre}}^{i}+\alpha_{\text{now}}^{i}*C_{\text{now}}^{i}*\left(1-O_{\text{pre}}^{d}\right)*I_{\text{post}}^{i}*I_{a}^{i},$$

where *C*_pre_^*i*^ denotes the R component of RGB value at current position of the intermediate image without consideration of indirect scattering; *C*_post_^*i*^ denotes that with indirect scattering term; *C*_now_^*i*^ denotes the R component value of current resampling voxel when computing the indirect scattering term.

**Figure 4:**
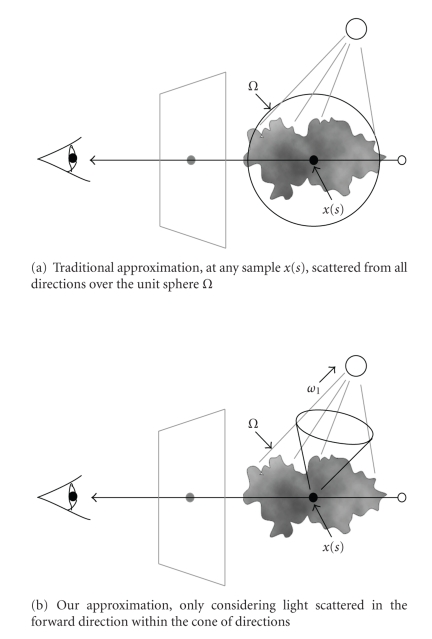
Indirect scattering approximation comparisons.

Considering each slice processed in sequence from forward to back in Shear-Warp algorithm, let *I*_*a*_^*i*^ in (29) be the average of six pixels which include the pixels at position *p* *o* *s* of indirect lighting memory of next or/current slice and their neighbor four pixels. Here, *I*_*a*_^*i*^ is the approximation value of $\sum_{p=1}^{n}\Delta \Phi_{p,i}\left(x,{\vec{\omega }}_{p}^{\prime }\right)/\left(4/3\right)\pi r^{3}$ in (12). The approximation of *I*_*a*_^*i*^ is different from traditional approximation [27]. In traditional approximation [27] of physically based light transport equation, incoming light, at any sample *x*(*s*) of general light transport scenario, scattered from all directions over the unit sphere *Ω* is considered as shown in Figure 4(a) and (12). Instead, the approximation of *I*_*a*_^*i*^ only considers light scattered in the forward direction within the cone of directions as shown in Figure 4(b), which is similar to document [2]. Reasons for the approximation of *I*_*a*_^*i*^ are as follows. (1) Each slice is processed in sequence from forward to back in this preintegrated Shear-Warp algorithm. (2) IVROM and its implementation ITVRM are hoped to be readily applied on a commodity PC. (3) According to [2], the question of whether the missing paths involving lateral movements outside the cone or any backscattering create a barrier to achieving important visual effects is an empirical one. Human viewers are believed not highly sensitive to the details of indirect volume lighting; so there is reason to hope that our approximation is useful. Here the minimum cone is used to approximately compute the effect of indirect scattering term instead of cone [2, 25].

The indirect scattering value of G and B components of the intermediate image can be calculated by the same way.

**Step 4**

Read all the data of indirect lighting memory of next slice to be processed into the memory of the current slice; then set all the indirect lighting memory of next slice as value 1.

**Step 5**

Process next slice.

### 4.4. Memory Consumption

The proposed ITVRM integrates the IVROM model, Shear-Warp, and preintegrated volume rendering algorithm; so it is similar to the Shear-Warp volume rendering. In the above implementation processes of the CPU-based ITVRM algorithm, the memory consumption of this ITVRM method is increased a little bit in comparison with that of the traditional Shear Warp algorithm [21]. As to this method, extra memories for the storage of the look-up table memory of the indirect opacity and preintegration, the direct lighting and indirect lighting memory of current slice, and indirect lighting memory of the next future slice are needed. That is, if the volume resolution is *o* ∗ *p* ∗ *q* with an intensity value of *m* bits (now an intensity value of CT slices is usually 12 bits), and the resolution of the largest intermediate image is *w* ∗ *h*, extra memories of (*w* ∗ *h* ∗ 3 + 2^*m*+1^) ∗ size of (float) are needed. Its increasing quantity is little if compared with the necessary memory for the storage of original mass 3D data set. So it can be concluded that the advantage of shear-warp algorithm over the texture-based is not canceled off by integrating lighting models.

## 5. Experimental Results

The algorithm is programmed in C++ language. The hardware environment of the host computer is Pentium4 CPU with frequency 3G, 1024 M RAM, and Graphic Card Geforce 6800 GT.

In order to investigate the effect and feasibility of the proposed optical model applied for translucent volume rendering of 3D medical image, the medical CT slices are reconstructed by both methods of ITVRM and traditional Shear-Warp volume rendering, respectively. The proposed ITVRM integrates the optical model IVROM, and the traditional Shear-Warp volume rendering uses traditional LAEM-BP. They are implemented under the same condition, that is, the same transfer function and view angle. For example, if setting the view angle *α* = −90° and *β* = 0°, that means the view light is rotated by −90° about *X* axis and 0° about *Y* axis. The medical CT slices used are data set of 512 ∗ 512 ∗ 377 with an intensity value of 16 bits.

**Figure 5:**
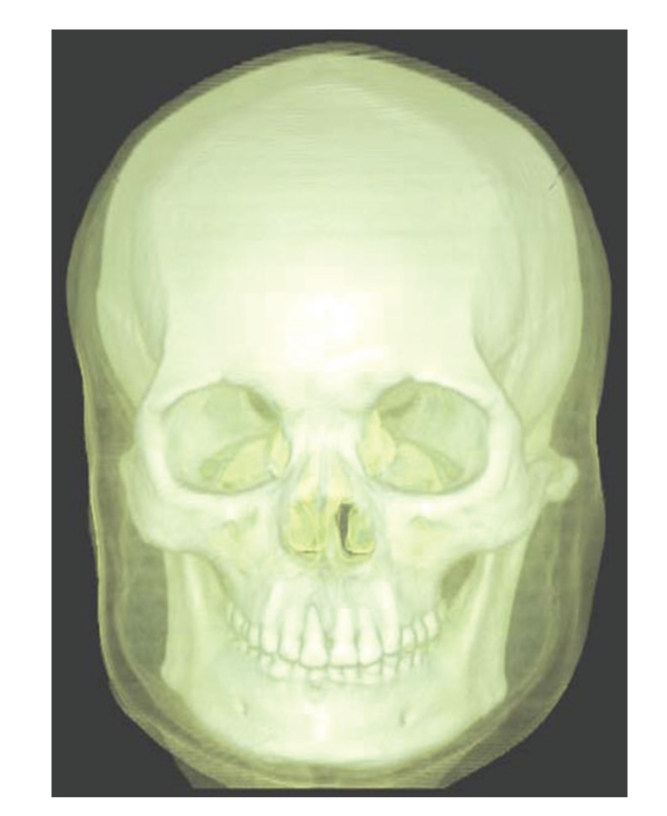
The result by the IVROM I.

**Figure 6:**
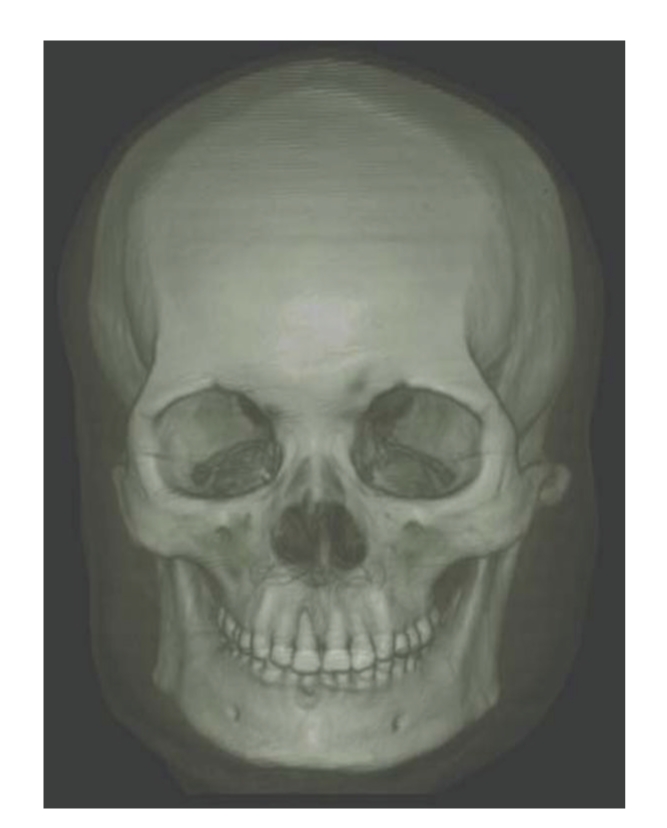
Direct scattering of IVROM.

**Figure 7:**
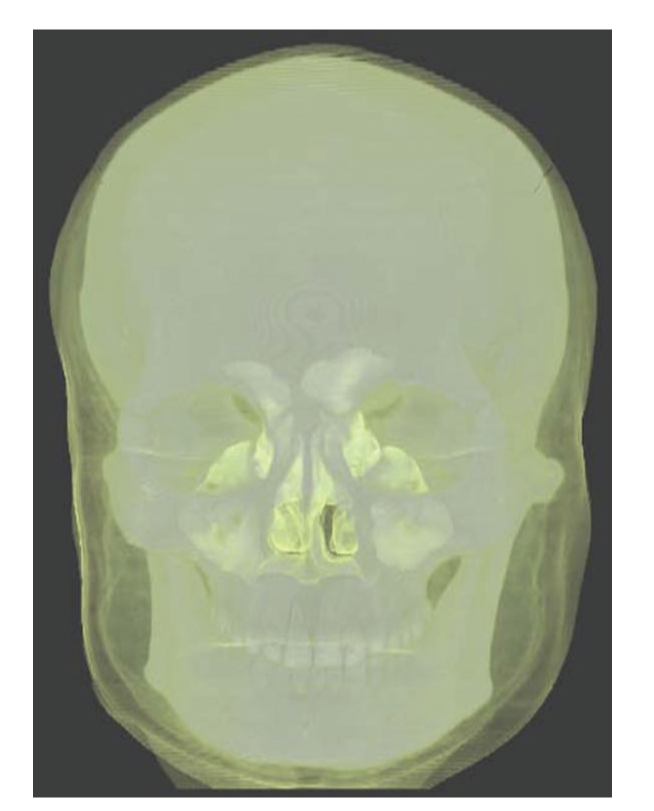
Indirect scattering of IVROM.

**Figure 8:**
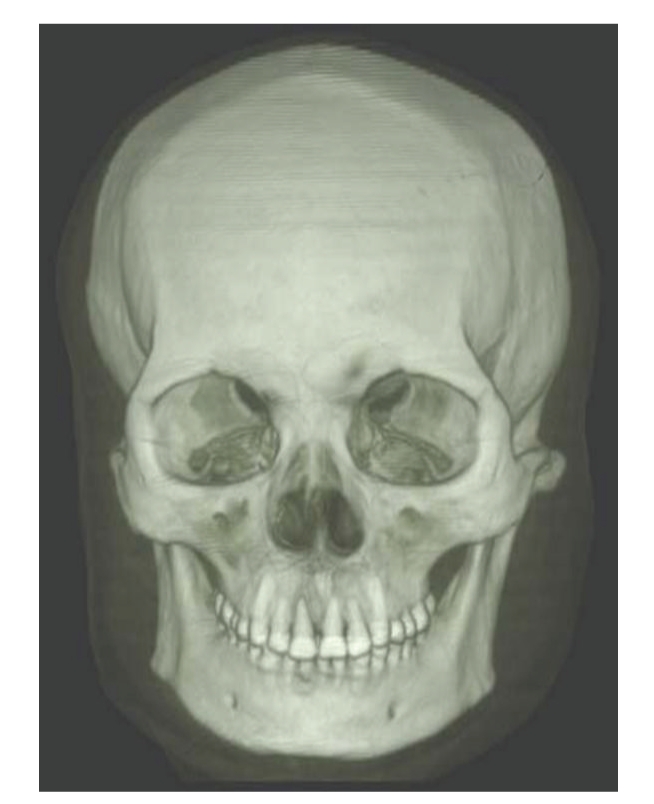
The traditional LAEM-BP model I.

**Figure 9:**
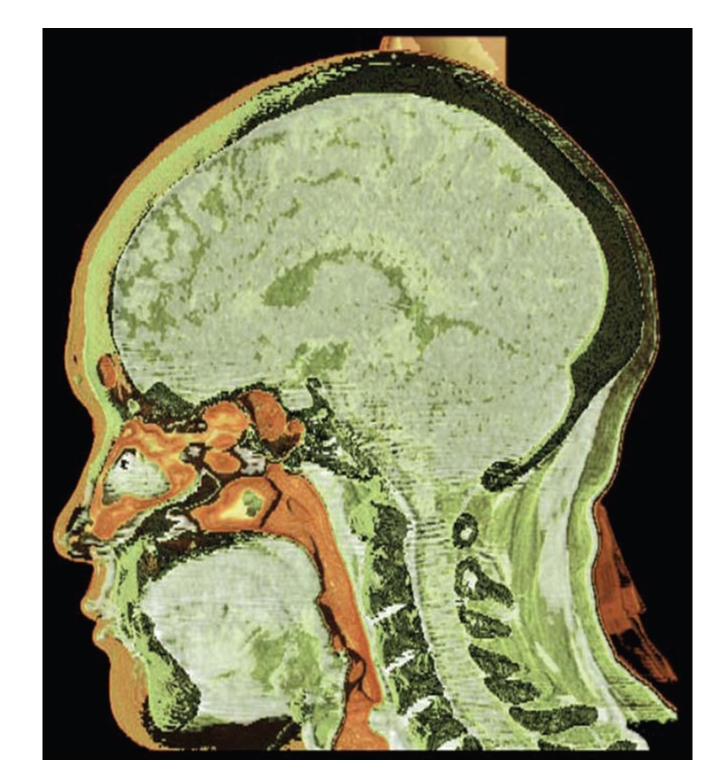
IVROM II.

**Figure 10:**
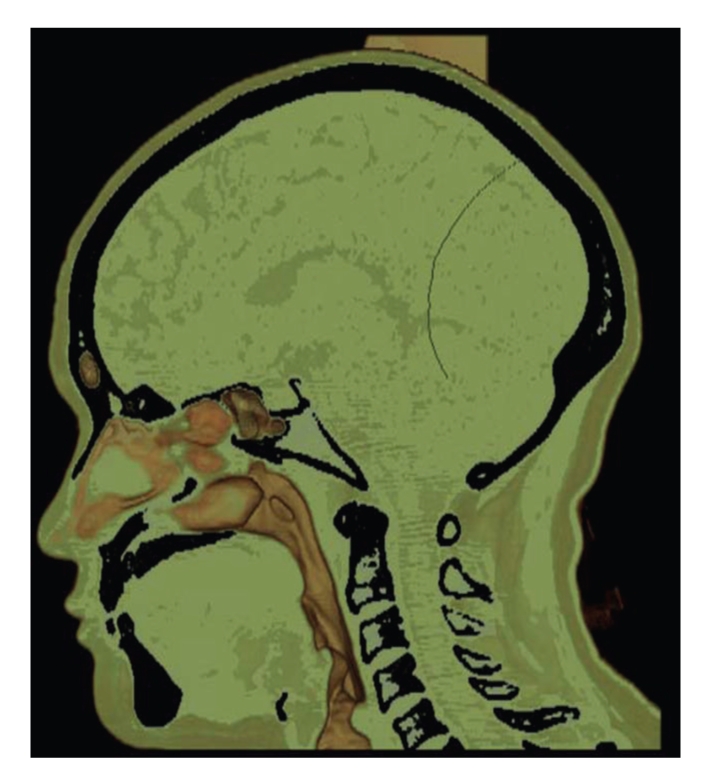
The traditional LAEM-BP model II.

Results are separately shown in Figures 5–10. Figure 5 is the result of ITVRM by optical model IVROM; Figure 6 is the result of direct scattering of IVROM; Figure 7 is the result of indirect scattering of IVROM; Figure 8 is the result of Shear-Warp volume rendering by traditional LAEM-BP. From the comparison between Figures 5 and 8, it can be obviously seen that the detailed information of inner structures and the inner hidden interfaces between different mediums can be displayed more clearly in Figure 5; thus the proposed method is more suitable for medical application, such as diagnosis.

Figures 9 and 10 show the 3D results of translucent volume rendering for human brain by the proposed ITVRM using optical model IVROM and the traditional Shear-Warp using LAEM-BP, respectively. Note that the soft tissue of the brain and the gradient norm difference of different tissues, such as cerebral white matter and ectocinerea, have nearly no difference between them. So the detailed information of the soft tissue of brain cannot be depicted clearly in Figure 10 which is reconstructed by traditional model LAEM-BP. Compared with Figure 10, it is obvious that the detailed information of inner structures and the inner hidden interfaces between different mediums can be displayed more clearly in Figure 9.

**Figure 11:**
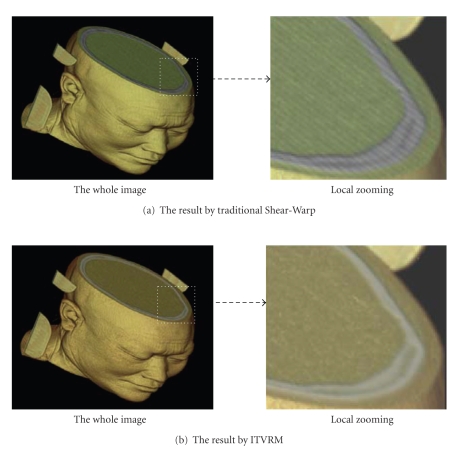
The reconstruction effect comparison between ITVRM and traditional Shear-Warp.

**Figure 12:**
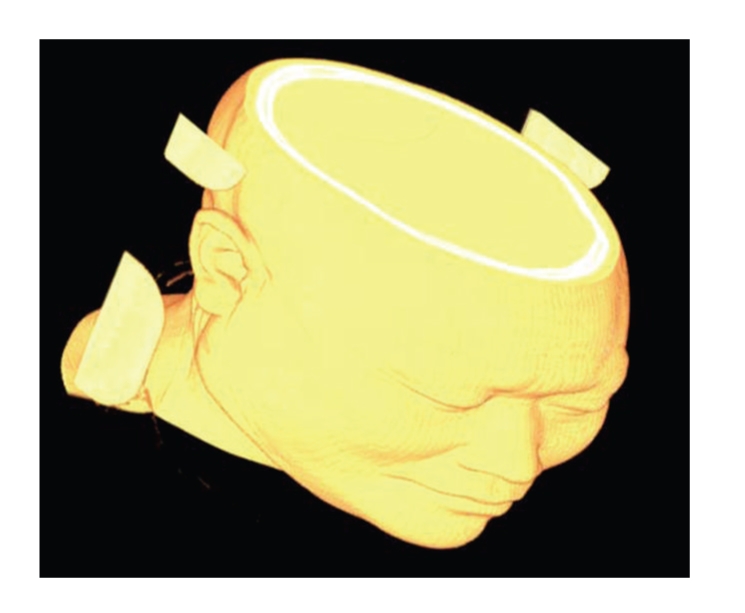
The result by preintegrated volume rendering without shading.

Figure 11 is the Reconstruction effect comparison between ITVRM and traditional Shear-Warp. Compared with Figure 11(a), it is obvious that the aliasing and staircase effects resulting from under-sampling in Shear-Warp are avoided by using the proposed ITVRM algorithm. Figure 12 shows the 3D results of volume rendering for human head by preintegrated volume rendering without shading and combining the proposed IVROM model. Compared with Figure 12, it is obvious that the detailed information of interfaces of the 3D medical image can be displayed more clearly in Figure 11(b).

The above experimental results verify that it is very effective to the proposed ITVRM method applying the presented optical model IVROM, compared with the LAEM-BP model; thus it is more valuable for medical application.

The comparison of runtime for 3D reconstruction between the proposed between ITVRM and traditional Shear-Warp is shown in Table 2. In this test, the condition is kept unchanged except the *β* is rotated by the increment of 30 degrees each step, so it has a total of 12 steps in a cycle, and then the running time is averaged. From Table 2, it can be seen that the running time for the proposed ITVRM using optical model IVROM is just a little longer than that for the traditional Shear-Warp using LAEM-BP model. The reasons can be explained that the calculation for direct and indirect lighting cumulation intensity, as well as the calculation of indirect scattering, needs more time than that of the traditional LAEM-BP model.

Besides, in the processing of setting transfer function, the runtime for translucent volume rendering method by the proposed ITVRM combining IVROM model is also a little longer than the traditional Shear-Warp combining LAEM-BP model. The reason is because that the execution of checking the look-up table for the weight of indirect opacity, direct scattering, and indirect scattering needs more time for the proposed optical model IVROM. From the above analysis, it can be drawn that even the running time for the proposed ITVRM combining IVROM model is longer than that of the traditional Shear-Warp combining LAEM-BP model, but it can be still realized on a commodity PC; moreover, the quality of the reconstructed 3D image of ITVRM combining IVROM is greatly improved.

## 6. Conclusions

In this paper, an improved volume rendering optical model (IVROM) for translucent volume rendering and its implementation using the Shear-Warp and preintegrated Volume Rendering algorithm are proposed in this paper, which can be readily applied on a commodity PC. In the proposed model, the lighting absorption and emission model are employed; moreover, the factors of volumetric shadowing, direct, and indirect emission are also considered. In addition, the realization of translucent volume rendering method ITVRM combining IVROM model, Shear-Warp, and preintegrated volume rendering algorithm is presented in detail. So the 3D medical image could be reconstructed efficiently, and the detailed information of inner structures, as well as the inner hidden interfaces between different mediums, can be display clearly. Experiment results demonstrate the good performance of the proposed method. Therefore, it is very preferable for practical applications.

## Figures and Tables

**Table 1:** Important terms used in the paper.

Symbol	Definition
*x*	Space coordinate of a voxel
*s*, *l*	Distance along the view direction
*x*(*s*)	3D coordinate along the view direction
$\vec{\omega }$	View direction
*ω*′	Ray direction
$I(\text{x},\vec{\omega })$	Intensity at position *x* along $\vec{\omega }$
*T*(*s*, *l*)	Attenuation from *x*(*s*) to *x*(*l*) along the view direction
*τ*	Attenuation coefficient
*R*(*x*)	Surface reflectivity color in position *x*
*B*_*s*_(*x*)	Value of Blinn-Phong surface shading model
*S*	Scattering lighting intensity
$r(\text{x},\vec{\omega },\omega^{\prime })$	A bi-directional reflection distribution function (BRDF).
*g*	Ray direction
*L*_*g*_	Intensity of light source
*l*_*g*_	Distance along ray direction
*d*	Direct scattering
*i*	Indirect scattering

**Table 2:** Comparison of reconstruction efficiency.

The used volume rendering method	Runtime of preprocessing(s)	Runtime(s)
The traditional shear-warp with LAEM BP mode	16.974	1.347
The proposed ITVRM combining IVROM and Preintegrated volume rendering	18.823	2.915
